# Current successes and remaining challenges in protein function prediction

**DOI:** 10.3389/fbinf.2023.1222182

**Published:** 2023-07-27

**Authors:** Constance J. Jeffery

**Affiliations:** Department of Biological Sciences, University of Illinois at Chicago, Chicago, IL, United States

**Keywords:** protein function, function prediction, protein structure, databases, structure and function

## Abstract

In recent years, improvements in protein function prediction methods have led to increased success in annotating protein sequences. However, the functions of over 30% of protein-coding genes remain unknown for many sequenced genomes. Protein functions vary widely, from catalyzing chemical reactions to binding DNA or RNA or forming structures in the cell, and some types of functions are challenging to predict due to the physical features associated with those functions. Other complications in understanding protein functions arise due to the fact that many proteins have more than one function or very small differences in sequence or structure that correspond to different functions. We will discuss some of the recent developments in predicting protein functions and some of the remaining challenges.

## 1 Introduction

During the past quarter century, advances in biochemistry, structural biology, proteomics, cell biology, genomics, and bioinformatics have yielded an explosion of information about protein functions. In addition to advancing our understanding of individual proteins, these advances have helped increase our understanding of macromolecular complexes, biochemical pathways, and the regulation of biological processes. This knowledge can be used to identify proteins and functions to target in developing novel therapeutics, for developing synthetic biological circuits or protein-based drug delivery systems, or for use in manufacturing and bioremediation. Our ability to predict protein functions has not kept up with the millions of protein sequences available from translating protein-coding genes from genome sequencing projects, as can be seen by the many protein entries listed as “predicted” or “probably” in the UniProt Knowledgebase (UniProt Consortium, 2021). This perspective article describes some of the recent successes in the field, the remaining challenges, and suggestions for directions needed for the future.

## 2 Successes

The critical assessment of functional annotation (CAFA) community challenge compares the successes of function prediction algorithms and has found significant improvements over the past 10 years (Radivojac et al., 2013; Jiang et al., 2016; Zhou et al., 2019). The results from the most recent challenges, CAFA2 and CAFA3, indicated that the most successful approaches used machine learning and sequence alignment and that integrating data from multiple complementary sources can improve the accuracy of predictions. Amino acid sequences, three-dimensional structures, protein or RNA expression profiles, genomic context, and molecular interaction data can all aid in improving function prediction. The organizers reported that the GOLabeler method performed significantly better than all other methods in the challenges. The method integrates the frequency of Gene Ontology (GO) terms, sequence alignments, patterns of amino acids, the presence of domains and motifs, and additional biophysical properties in a learning-to-rank (LTR) application of machine learning (You et al., 2018). A recent review by Bonetta and Valentino et al. (2023) describes more generally the machine learning approaches and techniques used for protein function prediction in the literature, including feature selections, algorithms and models, and implementation and evaluation.

Advances in bioinformatics and other computer-based algorithms have also helped make possible the discovery of vast amounts of information about protein functions from biochemical, biophysical, cell biology, and other experimental approaches. Advances in computational methods have improved the ability to determine cellular localization when using microscopy, identify gene neighbors in genome sequences (which indicates proteins might be involved in the same pathway or structure), identify changing levels of protein expression under different cellular conditions (for example, heat shock), or co-expression with other proteins (suggesting interaction in a biochemical pathway or multiprotein complex). Advances in bioinformatics have also aided in interpreting the results of proteomics studies, for example, in identifying protein fragments from mass spectrometry that help identify binding partners (proteins, small molecules, and sequences of DNA or RNA) and the presence of proteins in multiprotein complexes (for example, proteasomes and ribosomes), although a protein’s function in those locations and complexes, whether regulatory, scaffold/structural, or catalytic, is often unknown.

Advances in computational methods have also been important in determining or predicting protein structures, which can provide some information about a protein’s function. Advances in cryo-electron microscopy (Cryo-EM), X-ray crystallography, and NMR have yielded more protein structures and, importantly, structures of complexes with other proteins, DNA, RNA, and small-molecule ligands that provide key information about function. Recent successes in structure prediction using AI and homology modeling have yielded more models of protein structures that can also provide information about the overall fold; however, they do not always provide detailed information about active and interaction sites (Jumper et al., 2021). Comparisons of multiple protein structures can also help identify a structural class or a structural motif that can give a general idea of the function a protein might have.

Information about protein functions and other characteristics identified through diverse studies can be spread across many journal articles and other resources. Online databases are valuable tools that bring together this information. The UniProt Knowledgebase integrates and organizes information about protein sequences, structures, and functions for millions of proteins (UniProt Consortium, 2021) The Protein Data Bank contains over 100,000 experimentally determined protein structures (Berman et al., 2000). Some databases bring together information about specific types of proteins. For example, the Enzyme Portal is a database and tool for information and analysis of enzymes (Zaru et al., 2022). The MoonProt database is a collection of information about proteins that have been experimentally demonstrated to have more than one function (Chen et al., 2018). DisProt is a collection of information about proteins with regions of intrinsic disorder (Piovesan et al., 2017; Hatos et al., 2020). Collections of sequences and structures of many proteins with similar functions can provide training sets for developing predictive algorithms.

Advances in computational methods and projects for analyzing large quantities of protein structures, along with information about their functions, have been important for identifying features associated with specific functions. For example, the sub-classification of protein superfamilies into functional families (FunFams) and functional domains (Scheibenreif et al., 2019; Das et al., 2021) or the identification of constellations of amino acids in an enzyme’s active site related to catalytic function (Furnham et al., 2014; Riziotis and Thornton, 2022) can be used as the basis for developing novel or improved algorithms for predicting the functions of other proteins and in the development of novel insights regarding the functions of the classified proteins.

## 3 Current challenges

Many challenges remain in predicting protein functions, including the lack of characteristics or features correlated with some types of functions upon which to build an algorithm; the presence of many homologous proteins with small differences in sequence or structure that result in the proteins having different functions; the ability of proteins to have multiple functions; and a large number of proteins and types of proteins for which assays, representative structures, or other information about their functions are not known.

For many biochemical or biophysical functions, a sequence, structural motif, or other characteristic has not been identified that correlates with that specific function. For some types of functions, it is because the physical requirements of the function are only weakly conserved. For example, protein–protein interaction sites can consist of a relatively smooth region on the surface that is not well conserved, so predictions of the locations of pockets on the protein surface or comparisons of amino acid motifs or constellations are not as successful as predicting the locations of catalytic sites. Hundreds of proteins have been found to bind to RNA but do not contain any of the canonical RNA-binding domains, and for most of these proteins, it is not known which parts of the proteins interact with RNA. In addition, relatively small parts of a protein can be involved in a function. Plasminogen-binding site requirements for several proteins have been found to mainly involve a lysine at the protein terminus. Intrinsically disordered proteins or domains (IDPs) do not have a folded three-dimensional structure and also frequently use small sequences and/or short helices to interact with other proteins. In addition, when a sequence or structural motif is found that correlates with a function of the protein, it might only be one aspect of the function. For example, binding to DNA might be the main function of some proteins, whereas for other proteins, DNA binding is just one aspect of a larger function, such as binding to and then catalyzing the cleavage of the DNA. Similarly, the binding of a small molecule or ion such as Ca++ could be a mechanism for regulating the protein’s function, for example, in tropomyosin in muscle contraction. Some of the best predictors of protein characteristics are programs that predict the presence of transmembrane alpha helices, for example, TMHMM (Sonnhammer et al., 1998). These programs can predict approximately 25% of proteins that are transmembrane proteins with significant accuracy, but additional information is needed to determine the specific function, i.e., whether the protein acts as a channel, transporter, or receptor or functions as a structural attachment for the extracellular matrix or the cytoskeleton.

Another challenge in predicting protein function is that predictions based on sequence or structural homology can be inaccurate because even proteins with significant amino acid sequence identity can have different functions. Proteins with small changes in the amino acid sequence in or near the active site might not share the catalytic activity of other members of an enzyme family but instead might have a different function, such as a different catalytic mechanism or a different substrate. The enolase superfamily contains evolutionarily related enzymes with similar structures, a (β/α)7β-barrel (TIM-barrel) fold, and similarities in their active sites and catalytic mechanisms, but the proteins in different subgroups catalyze different reactions. The superfamily contains enolases, which convert 2-phosphoglycerate to phosphoenolpyruvate in glycolysis; muconate lactonizing enzymes, which break down aromatics derived from lignin into citric acid cycle intermediates; mandelate racemases that interconvert the (S)-mandelate and (R)-mandelate enantiomers; 3-methylaspartate ammonia lyases that break down L-threo-3-methylaspartate to mesaconate and ammonia; and other enzymes (Hasson et al., 1998; Schmidt et al., 2001; Gerlt et al., 2005; Gerlt et al., 2012). The aminotransferase family also contains paralogs that share certain amino acids in the active site, bind the same pyridoxal phosphate cofactor through covalent attachment to the sidechain of an active site lysine, and transfer an amino group from one substrate to another substrate. While some aminotransferases are specific for aspartate, others act on branched-chain amino acids or other substrates.

Many members of enzyme families have three-dimensional folds or domains that resemble catalytically active members of the family but lack catalytic activity altogether. These pseudoenzymes are found in most enzyme superfamilies, including pseudokinases, pseudoubiquitin ligases, and pseudonucleases. Relatively small changes, such as lacking key catalytic amino acids in the active site, result in a protein that does not have catalytic activity but is instead involved in another function, for example, regulating a catalytically active subunit or acting as a scaffold for bringing together a multiprotein complex. Some pseudoenzymes still bind to a canonical ligand or cofactor, but instead of catalysis, they have a role in signaling pathways, transcription, or translation [reviewed in the work of Eyers and Murphy (2016), Todd et al. (2002), Walden et al. (2018), Pils and Schultz (2004), Jeffery (2019), Adrain and Freeman (2012), Zettl et al. (2011), and Murphy et al. (2017a), Murphy et al. (2017b)].

Predictions of function are also complicated by moonlighting proteins, proteins that have two or more biochemical or biophysical functions (Jeffery, 1999; Jeffery, 2017). For example, a protein might have a catalytic function inside the cell while simultaneously performing another function on the cell surface, where it acts as an adhesin that binds other cells. The taxon-specific crystallins are enzymes that have a second function as structural proteins in the lens of the eye (Piatigorsky and Wistow, 1989). For example, lactate dehydrogenase is the epsilon crystallin in birds and crocodiles (Wistow et al., 1987; Hendriks et al., 1988). Some enzymes have a catalytic function and also a second function in which they bind to transcription factors or directly to DNA or RNA to regulate transcription or translation (Commichau and Stulke, 2015). Function prediction methods do not always find both functions. Some of the challenges include finding a true negative test set for developing predictive algorithms because even homologous proteins that lack one of the two functions of a moonlighting protein might have a different second function. There might also be many other functions that have not yet been identified. Even if multiple functions are found, there can still be additional functions that have not been found. Glyceraldehyde 3-phosphate dehydrogenase (GAPDH) is an enzyme in glycolysis that has also been found to be a DNA-binding protein and an RNA-binding protein and participates in multiple multiprotein complexes. GAPDH, like many other moonlighting proteins, appears to be a typical protein without unusual features that might suggest it has multiple functions, so many other proteins might also have multiple functions. Metamorphic proteins and morpheins, which have multiple three-dimensional structures (Jaffe, 2005; Porter and Looger, 2018; Dishman and Volkman, 2022), in some cases corresponding to different functions, are also difficult to predict. Other proteins might also have alternative structures with other functions, but often, a single protein fold has been determined experimentally or predicted computationally, so other structures and functions might be present but as yet unidentified.

More generally, there are still protein functions that have not been identified or characterized. Proteins might be found in a location in the cell or associated with a cellular structure, but their presence there is not understood. A gene knockout of a protein might affect multiple biochemical pathways or cellular processes, but the actual biochemical or biophysical function of the protein is still unknown. Biochemical assays of activity, such as for catalytic activity, might not be available and could be challenging to develop, for example, for functions that involve regulating the function of another protein, forming part of the structure of a larger complex, or a scaffold protein whose main function is bringing other proteins together. Functions found only in few species, in a small number of cell types, or expressed under specific conditions are likely to be poorly characterized or not identified. In addition, some types of proteins have not been as thoroughly characterized as others, in part because it is not clear if they have functions. For example, there is a growing awareness that many microproteins, proteins with fewer than 100 amino acids, can have regulatory, structural, or other functions (reviewed by Brunet et al., 2020).

Because structural features can be important for helping identify functions, the ability to predict function can be limited by the absence of structures for many proteins. Proteins might not be amenable to structural methods, especially if they might not crystallize, are too big for NMR, or are too small for Cryo-EM. It might be challenging to produce enough of a specific protein for structural studies, or the structure might be unstable when the protein is isolated from the cellular context. In general, “wet lab” methods for solving protein structures are far slower than the identification of protein sequences, so there are far more protein sequences than structures. Recent significant advances in AI have made possible predictions of structures for many proteins for which experimental structures are not available, but the overall protein fold is only one piece of the puzzle. Structures with bound ligands can be important for identifying the catalytic amino acid residues. The prediction of the structure of a single protein might not reveal its function within a multiprotein complex. Because many functions involve multiple conformations, structures with a single conformation might not reveal potential interaction surfaces.

## 4 Future directions

The combination of improvements in computational methods with the increasing amount of information from experimental methods has the potential to continue to improve protein function prediction. Computational methods can provide information about sequence and structural homology, motifs, constellations of key amino acid residues, locations in a genome, and fast comparisons to information in databases about well-characterized proteins. Experimental methods can add vital information about protein binding to substrates and cofactors; protein–protein interactions; catalytic activity; formation of multiprotein complexes; timing of expression and co-expression with other proteins; and binding to DNA, RNA, and other macromolecules. For many types of functions, high-throughput proteomics studies can be valuable for providing information about hundreds or even thousands of proteins at a time, including many proteins for which there was no prior prediction of a specific function. In addition, in the future, whether high throughput or not, novel assays will be needed to identify understudied types of functions.

For determining protein function from structures, there is a strong need for accurate, high-resolution structure information obtained through experimental methods—X-ray crystallography, Cryo-EM, and/or NMR. Recent AI methods can be used to create a testable model by predicting an overall protein’s three-dimensional fold, but a prediction of the fold alone is not often sufficient to accurately determine the function of the protein. As previously described, many proteins with a wide variety of functions can share a three-dimensional fold. Detailed information about the arrangement of amino acids in an enzyme’s active site is needed to predict ligand binding, specificity, and catalytic mechanism. In many cases, to correctly predict the catalytic function of an enzyme, the protein alone is insufficient, and structures with bound substrates and cofactors are needed. The functions of many enzymes and other types of proteins also involve multiple protein conformations and, in some cases, alternative folds of domains or subunits, so multiple structures are needed to learn about the function. Functions usually involve complexes with other proteins, DNA, RNA, or other macromolecules, and the detailed interactions between these molecules are key to the function, so the structures of these macromolecular complexes would yield information about how they interact with these other molecules. At the same time, the increased number of predicted structures can be valuable for developing testable hypotheses that can be addressed through further experiments.

Collaborations involving experts in bioinformatics with biochemical and biophysical experimentalists could enable projects to test predictions with experiments in an iterative way to add to our knowledge of confirmed protein functions and also improve predictive methods. The Enzyme Function Initiative (Gerlt et al., 2011) was a collaborative project for functional assignment for members of the enolase protein superfamily with an integrated sequence–structure–function-based approach. A multidisciplinary set of teams organized in superfamily/genome, protein, structure, microbiology, and computation and data/dissemination cores worked together to select targets, predict and test *in vitro* substrate specificities and catalytic activity, determine X-ray crystal structures, study the *in vivo* context of the enzyme function, and annotate the results. The most recent CAFA project also included both computational predictions and mutational screening in *Candida albicans* and *Pseudomonas aureginosa* to obtain information about proteins with roles in biofilm formation and motility (Zhou et al., 2019).

In general, biochemists, biophysicists, and structural biologists could work together with computational biologists to develop projects that yield improvements in predictive methods by considering questions such as the following.

What are some of the predictions of functions that can be tested through biochemical experiments (binding studies, site-directed mutagenesis, catalytic activity assays, etc.)? The experimentalists can help in identifying experiments to test the predictions and specific proteins that would be amenable to the needed experimental methods. What types of functions have been found experimentally that the current prediction methods tend to miss? What additional structural or functional data would help in providing the needed training set(s) for improving predictive algorithms? Which data should not be included in training sets for a specific algorithm? For example, are some of the structures not solved at a high enough resolution?

In summary, in recent years, protein function prediction methods have seen significant advances with increasingly accurate protein function predictions. Challenges remain in identifying some types of functions, especially functions that do not correspond to known sequences or structural motifs, functions that vary even in very similar protein structures, functions of very small or intrinsically disordered proteins, functions that have not yet been identified or characterized, and proteins with combinations of multiple functions. In the future, the rapidly increasing amount of diverse kinds of experimental data, in combination with advances in computational methods that make use of these data, is likely to continue to improve the accuracy of function prediction and its applicability to more kinds of protein functions.

## Data Availability

The original contributions presented in the study are included in the article/supplementary material; further inquiries can be directed to the corresponding author.
